# Supplementary material for: Enhanced biennial variability in the Pacific due to Atlantic capacitor effect
Source: Nat Commun. 2017 Mar 20;8:14887. doi: 10.1038/ncomms14887 (PMC5364382; doi:10.1038/ncomms14887)
Supplement: Supplementary Information — Supplementary Figures and Supplementary Table [file ncomms14887-s1.pdf]

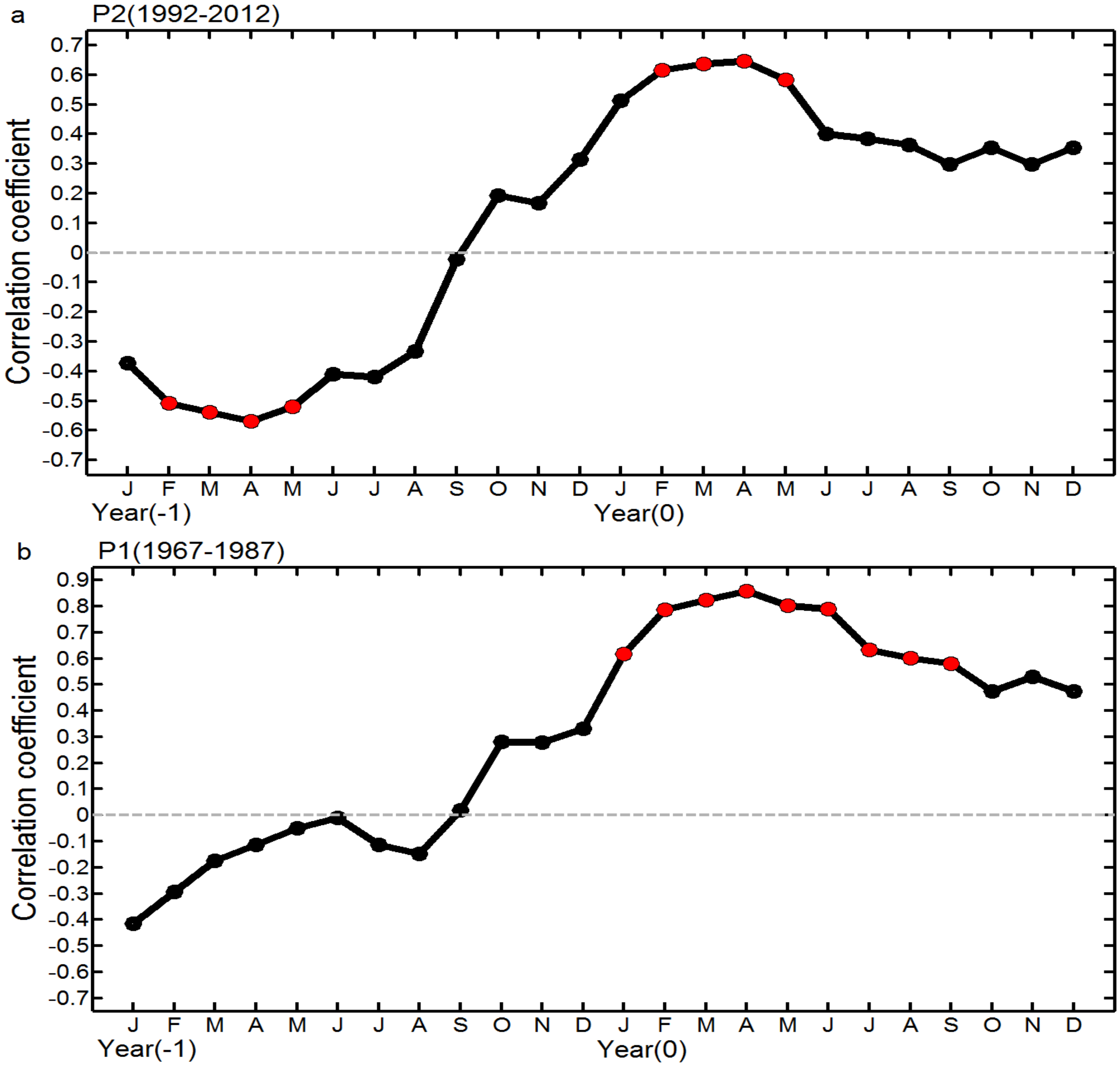

Supplementary Figure 1 | Correlations between the D(-1)JF(0) Nino 3.4 SST index and NTA SST index in each calendar months. Shown are for each calendar months from the ENSO developing year (Year(-1)) to the ENSO decaying year (Year(0)) during periods P2(a) and P1(b). The red dots represent correlations that are significant at the 95% confidence level.

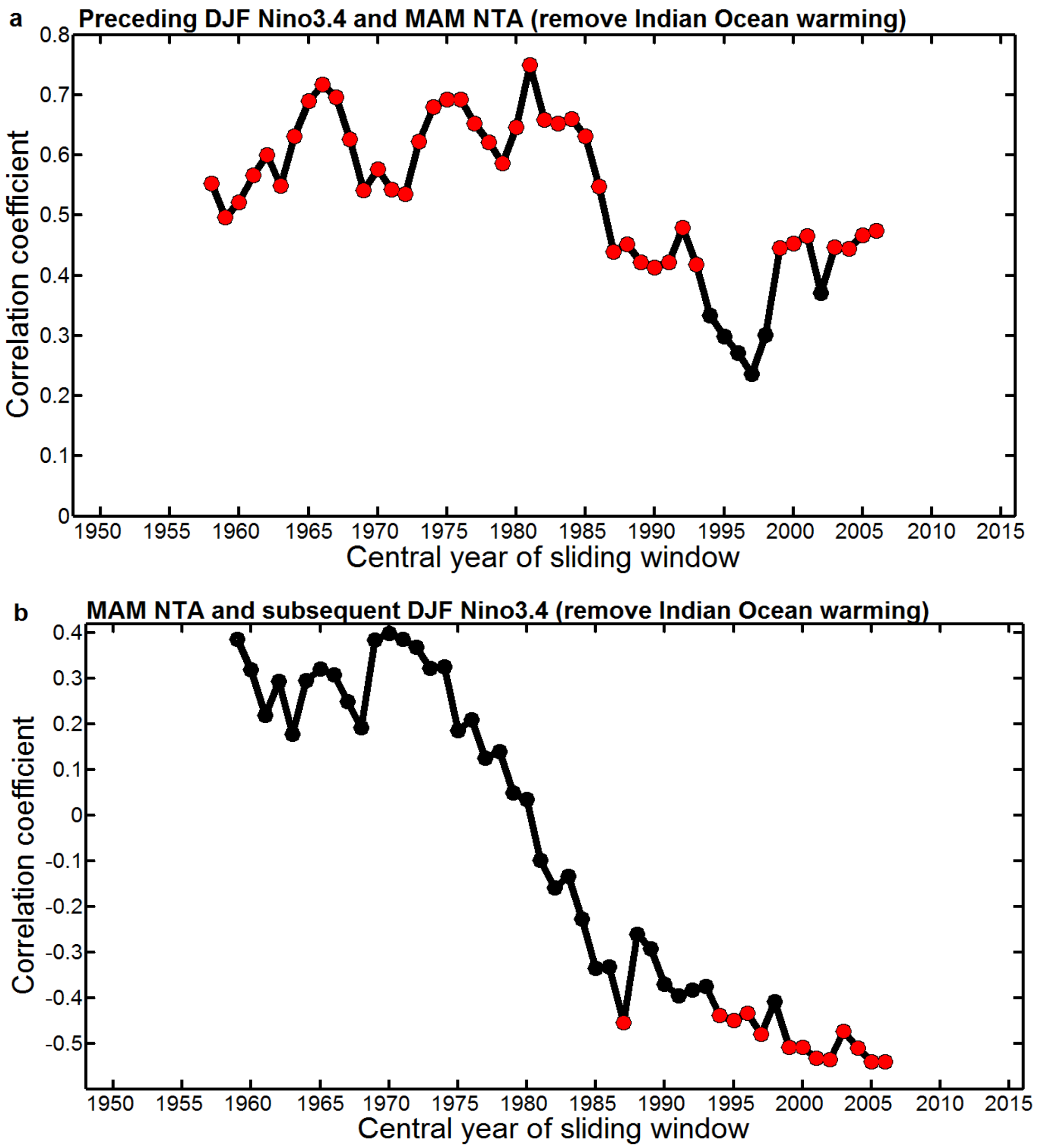

Supplementary Figure 2 | Same as Figure 1 but for partial correlation coefficients between MAM NTA SST and DJF Niño3.4 with the influences of the MAM Indian Ocean Basin index being removed. a. Correlation between preceding DJF Niño3.4 and MAM NTA SST. b. Correlation between MAM NTA SST and subsequent DJF Niño3.4. Red dots denote the correlation coefficients are statistically significant at the 95% level.

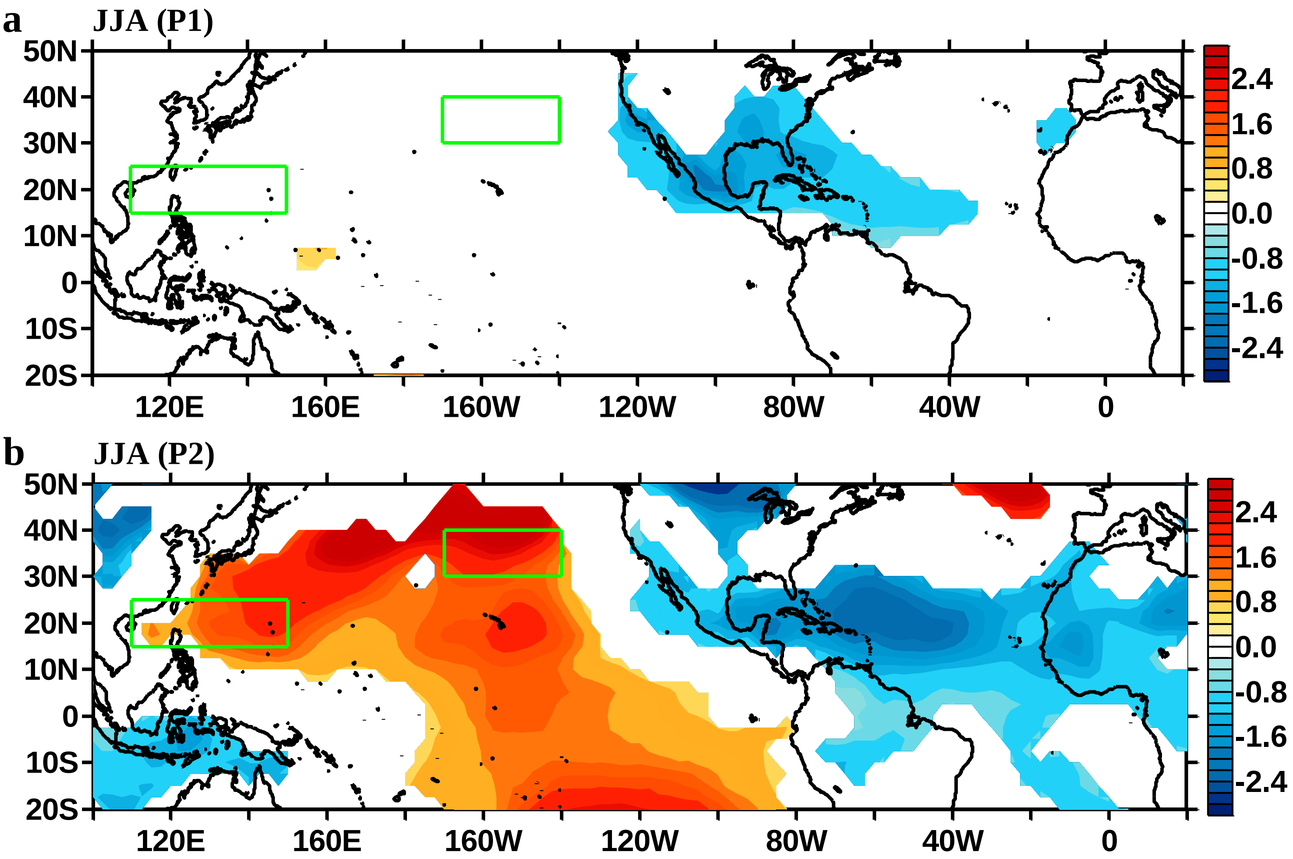

Supplementary Figure 3 | Regression of summer SLP with respect to spring NTA SST. a-b, Regressions of JJA SLP (shading, hpa) with respect to the MAM NTA SST during P1 **(a)** and P2 **(b)**, after excluding the impact of Niño3.4 SST during the previous DJF season. Only the values at the 90% confidence level or higher are shown. The two green boxes represent the domain for defining the WPSH and NPSH.

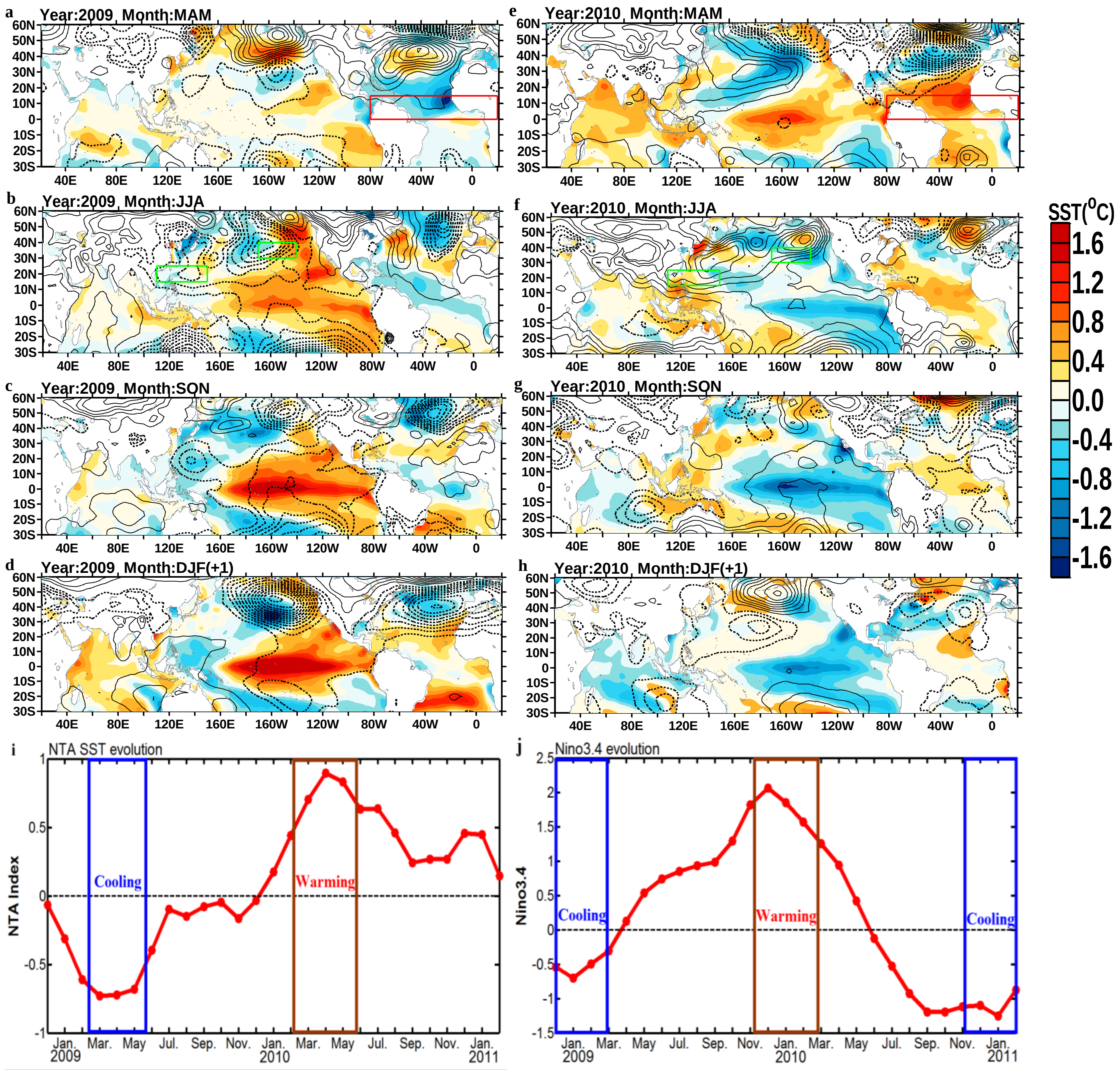

Supplementary Figure 4 | The biennial cycle during the 2009/10 El Niño event associated with the Atlantic-Pacific coupling. a-h, Seasonal evolutions of the SST anomalies (color) and SLP anomalies (contour, solid for positive and dashed for negative values) during the 2009/2010 event. **i-j,** Monthly time series of the NTA SST index (i) and Niño3.4 index (j). The red boxes (a,e) represent the domain to define the NTA SST index. The green boxes (b,f) show the domain to define the summer WPSH and NPSH indices.

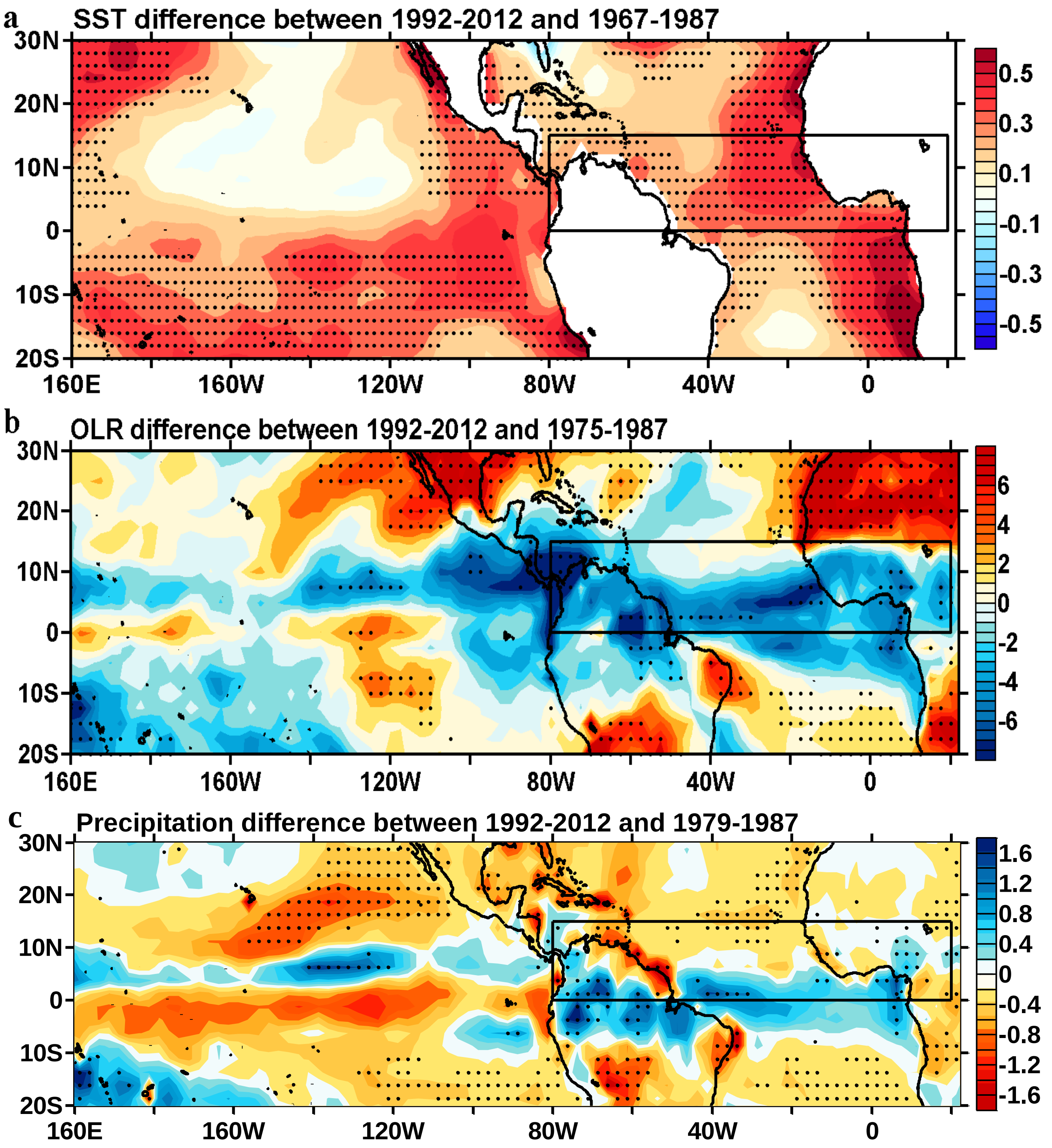

Supplementary Figure 5 | Differences between the mean background state before and after the early 1990s. Differences are shown for **(a)** MAM SST(℃), (**b**) MAM OLR (W m^{-2}), and (**c**) MAM precipitation (mm day^{-1}). Signals above the 90%-confidence level are highlighted by black dots. Due to the length limitation of available data in OLR and precipitation, the whole period during P1 was not fully covered in (b) and (c). The black box represents the domain of NTA.

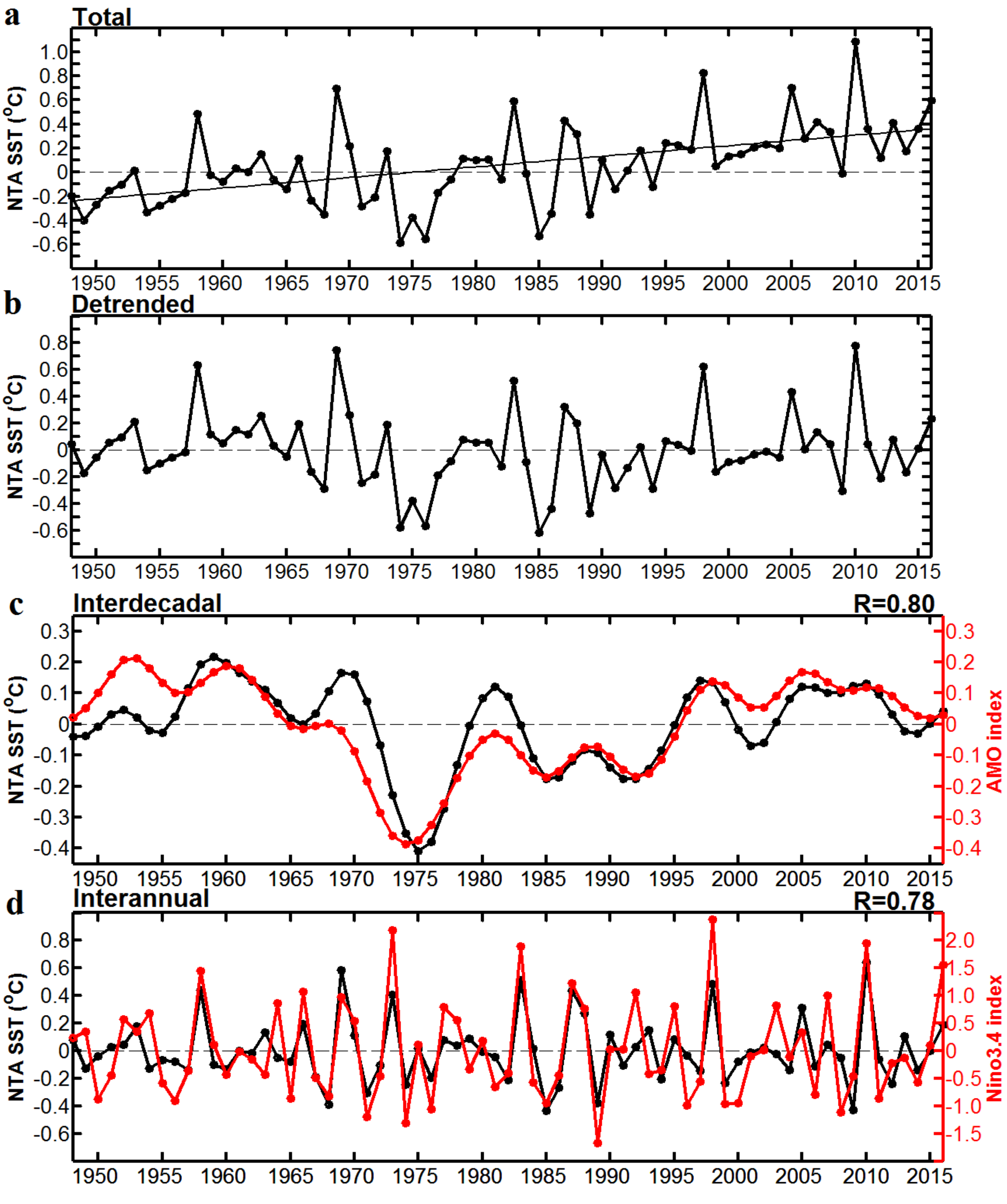

Supplementary Figure 6 | Time series of spring NTA SST during 1948-2016. Shown are **(a)** the total, **(b)** detrended (removing the linear trend), **(c)** interdecadal, and **(d)** interannual variations. The red line is for the spring AMO index in (c) and preceding winter Niño3.4 index in (d). The interannual (interdecadal) variability was obtained by applying a 7-yr high-pass (low-pass) Lanczos filter to the detrended anomalies. The correlation coefficients between NTA SST and AMO index/Niño3.4 index were shown at the right above panel in (c) and (d). The solid straight lines in (a) are the linear trend fitted to the raw time series.

Supplementary Table 1 | Correlation coefficients among the winter Niño3.4 index, PNA index, Atlantic Hadley circulation index and NTA SST index during the whole period, P1 and P2. The bold represents above the 95% confidence level. All examined correlation coefficients are significant at the 95% interval, except for the correlation between winter PNA index and NTA SST index during P1 which is only significant at the 90% interval.

	**Whole period (1948-206)**	**P1 (1967-1987)**	**P2 (1992-2002)**
Winter Niño3.4 and PNA index	**0.60**	**0.54**	**0.81**
Winter Niño3.4 and Atlantic Hadley circulation index	**-0.58**	**-0.69**	**-0.78**
Winter PNA index and NTA SST index	**0.46**	0.39	**0.70**
Winter Atlantic Hadley circulation index and NTA SST index	**-0.64**	**-0.67**	**-0.81**
Winter NTA SST index and spring NTA SST index	**0.70**	**0.67**	**0.75**
